# Steroid-Refractory Adult-Onset Still’s Disease: A Case Report

**DOI:** 10.7759/cureus.115661

**Published:** 2026-09-02

**Authors:** Pyae Phyo Kyaw, Thapye Mon, Pyae Phyo Thu, Aye Nyein

**Affiliations:** 1 Acute Medicine, Heartlands Hospital, Birmingham, GBR; 2 Internal Medicine, Heartlands Hospital, Birmingham, GBR; 3 Pulmonology, Queen Elizabeth Hospital Birmingham, Birmingham, GBR

**Keywords:** adult-onset still’s disease, biological therapy, fever of unknown origin, hyperferritinemia, interleukin-6

## Abstract

Adult-onset Still's disease (AOSD) is one of the systemic autoinflammatory disorders characterised by an evanescent salmon-pink rash, high spiking fevers, and inflammatory arthritis. AOSD is driven by dysregulated immune activation involving excess pro-inflammatory cytokines, specifically IL-1β and IL-18. Current consensus recognises AOSD and systemic juvenile idiopathic arthritis as a continuous disease spectrum. Because it lacks a definitive biomarker and mimics other severe conditions, AOSD remains a diagnosis of exclusion; early recognition is paramount to prevent complications including macrophage activation syndrome and haemophagocytic lymphohistiocytosis. We report the case of a 23-year-old man presenting with persistent pyrexia of unknown origin, an evanescent rash, inflammatory monoarthritis, marked neutrophilic leukocytosis, and profound hyperferritinaemia. Following an extensive multidisciplinary investigation to exclude connective tissue disease, malignancy, and infection, the patient successfully fulfilled the Yamaguchi classification criteria for AOSD. This case highlights the inherent diagnostic challenges associated with Still's disease. It demonstrates the critical importance of early multidisciplinary assessment and the timely escalation to targeted cytokine-directed therapies to achieve disease remission.

## Introduction

Initially described as the adult counterpart to systemic juvenile idiopathic arthritis (sJIA), adult-onset Still's disease (AOSD) poses a significant diagnostic challenge due to its heterogeneous presentation. The condition is classically identified by the triad of high-grade fevers, inflammatory arthritis, and an evanescent salmon-pink maculopapular rash [1], but often clinically resembles severe infections or hidden malignancies [1,2]. Underlying pathophysiology includes dysregulation of the innate immune system, with excessive production of interleukin (IL)-1β and IL-18 leading to a cytokine storm [2]. The lack of a definitive serological biomarker emphasises the need for prompt clinical recognition to prevent complications such as macrophage activation syndrome (MAS) and haemophagocytic lymphohistiocytosis (HLH) [2].

The aetiology of AOSD and its underlying pathogenetic mechanisms remain unknown. No definitive risk factors have been identified; however, environmental triggers are suspected. Several infectious agents have been reported in association with disease onset, including Epstein-Barr virus, cytomegalovirus, human immunodeficiency virus, Coxsackie virus, hepatitis A, B, and C viruses, and Mycoplasma pneumoniae [3,4]. Prevalence and incidence data are difficult to determine because of the broad, non-specific clinical presentation of AOSD. The estimated prevalence is greater than 1 per 100,000 population, with a slight female predominance [4,5].

The current understanding of sJIA and AOSD has shifted towards recognising them as age-related manifestations of the same disease spectrum rather than distinct disorders. Comparative studies and systematic reviews have demonstrated highly similar clinical and laboratory features between the two conditions, including recurrent spiking fever, evanescent rash, inflammatory arthritis, neutrophilic leukocytosis, elevated inflammatory markers, hyperferritinaemia, and the risk of MAS. Reported differences, such as the increased frequency of sore throat, myalgia, and weight loss in adults, are likely influenced by age-related symptom reporting and variations in diagnostic criteria rather than fundamental biological differences. These findings support the concept of a continuous disease spectrum. Recent international recommendations further recognise sJIA and AOSD as a single disease entity, termed Still's disease, promoting a unified approach to diagnosis, classification, and management across paediatric and adult populations [1,2].

In this report, we present the case of a 23-year-old man who exhibited pyrexia of unknown origin, inflammatory monoarthritis, an evanescent rash, marked neutrophilic leukocytosis, and profound hyperferritinaemia. After thorough investigation to exclude infection, malignancy, and connective tissue disease, he met the Yamaguchi classification criteria for AOSD [6]. This case specifically illustrates the diagnostic complexity of Still's disease and centres on the need for early multidisciplinary evaluation and prompt initiation of cytokine-directed therapy to facilitate disease remission.

The diagnosis of AOSD remains challenging because no disease-specific diagnostic biomarker exists, and clinical manifestations frequently overlap with severe infection, malignancy, and autoimmune disorders. Therefore, diagnosis relies on the recognition of characteristic clinical features, exclusion of alternative diagnoses, and application of established classification criteria [1,2,6]. In this case, both the Yamaguchi and Fautrel's criteria [7] were considered; however, the diagnosis was primarily established using the Yamaguchi criteria following an extensive negative investigation for infectious, malignant, and autoimmune causes of systemic inflammation [6].

Yamaguchi criteria

Diagnosis requires five or more criteria, of which two or more must be major. Major criteria include fever greater than 39°C lasting one week or longer, arthralgia or arthritis lasting two weeks or longer, a typical rash, and leukocytosis greater than 10,000/mm^3^ with greater than 80% polymorphonuclear cells. Minor criteria include sore throat, recent development of significant lymphadenopathy, hepatomegaly or splenomegaly, abnormal liver function tests, and negative tests for antinuclear antibody and rheumatoid factor. Exclusion criteria comprise infections, malignancies (mainly malignant lymphoma), and other rheumatic diseases (mainly systemic vasculitis).

Fautrel’s criteria

Diagnosis requires four or more major criteria, or three major and two minor criteria. Major criteria consist of spiking fever of 39°C or higher, arthralgia, transient erythema, pharyngitis, polymorphonuclear cells at 80% or higher, and glycosylated ferritin at 20% or lower. Minor criteria include a maculopapular rash and leukocytosis of 10,000/mm^3^ or higher.

Our patient’s clinical presentation closely matched the Yamaguchi criteria for AOSD. Following extensive exclusion of infectious, malignant, and autoimmune causes of persistent inflammation, he fulfilled the required diagnostic threshold, meeting four major criteria and additional supportive features. The patient demonstrated persistent fever ≥39°C lasting more than one week, inflammatory arthralgia/arthritis lasting more than two weeks, a typical transient evanescent rash associated with fever spikes, and marked neutrophilic leukocytosis (>10 × 10⁹/L with >80% neutrophils). Additional supportive findings included abnormal liver function tests and negative rheumatoid factor. Although antinuclear antibody (ANA) was positive at a titre of 1:1600, the absence of disease-specific autoantibodies, normal complement levels, and lack of clinical features suggestive of connective tissue disease supported the diagnosis of AOSD. Overall, the combination of characteristic clinical features, profound systemic inflammation, extreme hyperferritinaemia, and exclusion of alternative diagnoses demonstrated a strong match with the Yamaguchi classification criteria [6].

Due to its heterogeneous presentation and the absence of a definitive diagnostic test, AOSD remains a diagnosis of exclusion and is frequently mistaken for infection, malignancy, or other autoimmune diseases, often resulting in delayed diagnosis and treatment [1,5]. Although corticosteroids remain the cornerstone of initial therapy, a proportion of patients exhibit refractory disease requiring early escalation to targeted biologic agents [8]. We present a case of AOSD that highlights the diagnostic challenges associated with prolonged pyrexia of unknown origin and demonstrates the importance of timely recognition and escalation to interleukin-targeted therapy following an inadequate response to corticosteroids [8].

This article was previously presented as a case representation at the Society of Acute Medicine (SAM) conference on June 15, 2026. 

## Case presentation

A 23-year-old male, with a background of cerebral palsy and learning disability, presented to the emergency department on Day 1 with a two-day history of severe left knee pain along with high-grade fever. Apart from that, there were no other systemic symptoms such as respiratory, gastrointestinal, or neurological symptoms. There was no preceding history of gastrointestinal illness, genitourinary symptoms, or recent unprotected sexual encounters. Cardiovascular, respiratory, and neurological examinations were unremarkable. Examination of the abdomen demonstrated no hepatomegaly, splenomegaly, or ascites. There was no palpable peripheral lymphadenopathy. Musculoskeletal examination revealed a tender and swollen left knee without deformity or restriction of movement. The left ankle was also swollen with mild tenderness.

Initial blood tests demonstrated a marked increase in inflammatory markers, including a C-reactive protein (CRP) of 241 mg/L and neutrophil leukocytosis (Table 1). Sequential investigations were undertaken to identify the underlying cause. An X-ray of the left tibia and fibula first excluded acute fracture or bony abnormality. Orthopaedic assessment led to empirical treatment for septic arthritis with flucloxacillin and gentamicin, accompanied by knee joint aspiration. The synovial culture was negative and no crystals were detected. Three days later, magnetic resonance imaging (MRI) of the left knee (Figure 1) revealed a small joint effusion, but no evidence of osteomyelitis or septic arthritis. Despite these investigations, the patient continued to experience pain and swelling in the knee joint along with intermittent temperature spikes for more than a week.

**Table 1:** Summary of laboratory findings CRP: C-reactive protein; Hb: haemoglobin; WBC: white blood cell count; ESR: erythrocyte sedimentation rate; eGFR: estimated glomerular filtration rate; AST: aspartate aminotransferase; ALT: alanine aminotransferase; ALP: alkaline phosphatase; ANA: antinuclear antibody; ENA: extractable nuclear antigen; ANCA: antineutrophil cytoplasmic antibody; RF: rheumatoid factor; C3: complement component 3; C4: complement component 4; anti-dsDNA: anti-double-stranded DNA; IgG: Immunoglobulin G; IgA: Immunoglobulin A; IgM: Immunoglobulin M

Date	Day 1	Week 1	Week 2	Week 3	Reference range
CRP	241	421	248	137	0-5 mg/L
Hb	114	92	85	68	130-171 g/L
WBC	13.85	24.76	39.07	23.52	3.26 – 11.2 x 10⁹/L
Neutrophils	11.56	22.27	34.19	18.85	1.5-7 x 10⁹/L
Lymphocytes	1.69	1.81	3.52	3.44	0.5-4 x 10⁹/L
Platelets	203	252	418	252	150-400 x 10⁹/L
ESR	-	-	-	102	2-10 mm in 1^st^ hour
Creatinine	74	84	67	72	64-104 µmol/L
eGFR	>90	>90	>90	>90	90 - 150 ml/min/1.73m^2^
Bilirubin	-	8	7	19	<21 mmol/L
AST	-	-	28	-	11-34 IU/L
ALT	-	61	189	1048	0-55 IU/L
ALP	-	158	226	306	30-130 IU/L
Ferritin	-	-	>33511	>33511	22-501 ng/ml
Triglycerides	-	-	1.3	-	<1.7 mmol/L
Fibrinogen	-	-	6.6	-	1.5-4.1 g/L
ANA	-	-	Positive (1:1600)	-	-
ENA	-	-	Negative	-	-
ANCA	-	-	Negative	-	-
RF	-	-	Negative	-	-
C3	-	-	1.63	-	0.75-1.65 g/L
C4	-	-	0.28	-	0.14-0.54 g/L
Anti-dsDNA	-	-	9	-	0.6-9.9 IU/ml
IgG	-	-	14.9	-	6-16 g/L
IgA	-	-	2.7	-	0.8-2.8 g/L
IgM	-	-	1.1	-	0.5-1.9 g/L

**Figure 1:**
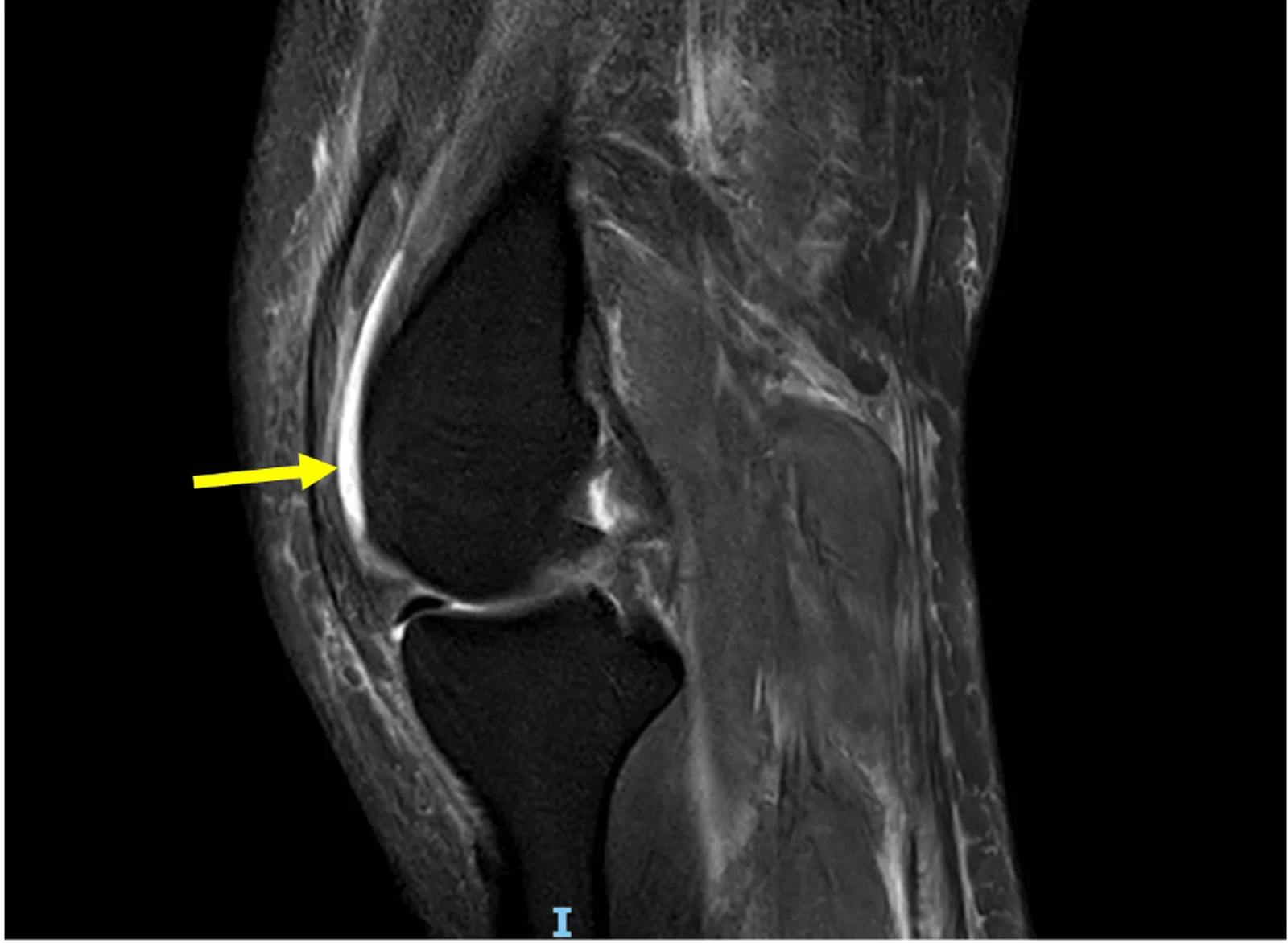
MRI of the left knee revealed a small joint effusion (as denoted by the yellow arrow) without evidence of osteomyelitis or septic arthritis

Additionally, he developed a transient erythematous rash affecting the upper limbs, particularly during fever spikes. The rash was short-lived and resolved spontaneously before it could be photographed or reviewed by the dermatology team. Inflammatory markers remained elevated, with a CRP of 421 mg/L, erythrocyte sedimentation rate (ESR) of 102, a peak white blood cell count of 39 × 10⁹/L, and neutrophils of 34 × 10⁹/L (87.2%), while lymphocyte counts remained within the normal range (Table 1). Antibiotics were escalated to piperacillin-tazobactam, which was later changed to meropenem.

We conducted extended investigations for the pyrexia of unknown origin. Blood cultures, urine cultures, and the respiratory viral BioFire panel were negative. Blood-borne virus screening, toxoplasma, parvovirus, Strongyloidiasis, Epstein-Barr virus, and cytomegalovirus serology were all negative. Computed tomography of the chest, abdomen, and pelvis with contrast revealed no source of sepsis, malignancy, lymphadenopathy, or splenomegaly. Transthoracic echocardiography excluded infective endocarditis.

The patient developed elevated alanine aminotransferase (ALT) and alkaline phosphatase (ALP) seven days after symptom onset (Table 1). Two weeks later, serum ferritin was markedly elevated at >33,511 ng/ml (Table 1). Simultaneously, autoimmune investigations were done because of persistent fever, joint pain, and transient rash. The immunology profile demonstrated a positive ANA titre of 1:1600, and rheumatoid factor (RF), extractable nuclear antigen (ENA), antineutrophil cytoplasmic antibody (ANCA), anti-double-stranded DNA (anti-dsDNA) antibodies, and complement levels (C3 and C4) were all within normal limits (Table 1). After excluding malignancy, infection, and other autoimmune diseases, the patient was diagnosed with AOSD as he fulfilled at least five Yamaguchi criteria, including fever lasting over one week, arthralgia for more than two weeks, a non-pruritic rash, leukocytosis with >80% neutrophils, abnormal liver function tests, and negative RF.

Clinical course and management

During the second week after presentation, the patient was diagnosed with AOSD and commenced on prednisolone 30 mg once daily for a week, following which the dose was gradually tapered. However, he developed recurrent fever and arthritis with knee effusion, and prednisolone was therefore increased to 50 mg once daily during week 4. Methotrexate was initially planned as a steroid-sparing agent; however, it was not initiated due to progressive deterioration in liver function, with ALT increasing to >1,000 U/L. Following multidisciplinary team discussion, the decision was made to commence subcutaneous anakinra 100 mg daily. While awaiting availability of anakinra, prednisolone 50 mg once daily was continued. Subsequently, anakinra was started in week 6, after which prednisolone was gradually tapered over six weeks to 5 mg once daily.

Outcome and follow-up

Three months after starting anakinra, the patient demonstrated significant clinical improvement, with complete resolution of fever, arthritis, and joint effusion. Inflammatory markers markedly improved, with ESR to 15 mm/hour, ferritin to 160 ng/ml, and CRP decreasing to 9 mg/L (Figure 2). 

**Figure 2:**
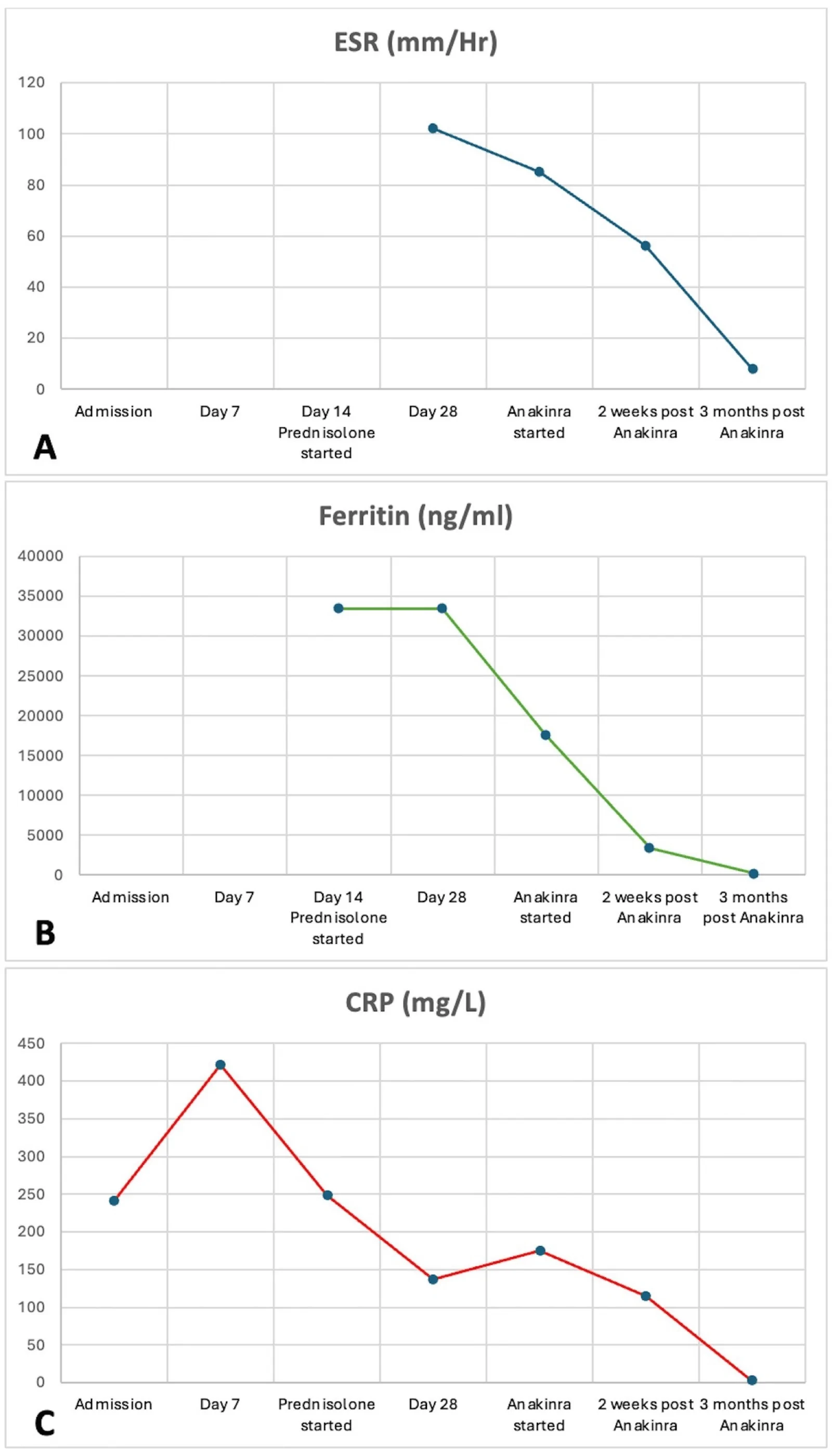
Trend of inflammatory markers over the course of treatment (A) ESR trend during treatment with prednisolone and anakinra and subsequent follow-up. (B) Ferritin trend during treatment with prednisolone and anakinra and subsequent follow-up. (C) CRP trend during treatment with prednisolone and anakinra and subsequent follow-up. ESR: erythrocyte sedimentation rate; CRP: C-reactive protein

Liver function tests also improved substantially, with ALT declining to 105 IU/L. He will continue to be followed up by the rheumatology team and is currently awaiting initiation of tocilizumab for greater convenience with weekly dosing rather than daily subcutaneous anakinra injections.

## Discussion

AOSD is an uncommon systemic inflammatory condition, with a reported global incidence of 0.16-0.62 per 100,000 people. It exhibits a bimodal age distribution, with peak onset occurring between 15-25 and 36-46 years. Importantly, AOSD poses a significant diagnostic challenge due to its non-specific presentation, which can closely mimic infections, malignancies, and other autoimmune conditions. Early and accurate diagnosis is crucial, as delayed recognition may result in adverse outcomes. Recent consensus by the European League Against Rheumatism (EULAR) and Pediatric Rheumatology European Society (PReS) recognises that AOSD and sJIA represent the same disease spectrum occurring at different ages, unified under the single term "Still's disease" [1,2].

While sJIA and AOSD are now recognised as a single disease continuum, distinct age-related phenotypic variations exist in their clinical presentation. A recent multicenter study observed that, compared to pediatric populations, adult-onset patients exhibit a significantly higher frequency of classic symptoms such as fever, evanescent rash, and myalgia [2]. Furthermore, laboratory abnormalities, particularly marked neutrophilia and elevated transaminases, are notably more prevalent in the adult phenotype. Our 23-year-old patient perfectly mirrored this aggressive adult phenotype. He presented with classic high-grade fevers and a transient erythematous rash, accompanied by profound neutrophilic leukocytosis (peaking at 34.19 × 10⁹/L) and severe hepatic involvement, with ALT surging to 1,048 IU/L [9,10]. Because profound hyperferritinaemia is a hallmark of both severe AOSD and its most dreaded autoinflammatory complications, HLH and secondary MAS were actively considered. However, the patient's HScore was calculated at 83 points, corresponding to a less than 1% probability of HLH [11]. Recognising this specific, high-risk adult phenotype is clinically vital, as severe transaminitis often portends a steroid-refractory course that necessitates early transition to targeted biologic therapies [8].

The pathophysiology of AOSD is characterised by a multisystemic autoinflammatory response driven by a profound imbalance between the innate and adaptive immune systems [3,4]. The process begins with an interaction between genetic susceptibility-specifically HLA-DRB1 polymorphisms (such as DRB1-12 and DRB1-15) and non-HLA gene variations like MEFV, NOD2, and CARD8-and environmental triggers, including viral (EBV, CMV) or bacterial (Yersinia, Mycoplasma) infections. The innate immune system acts as the main driver, where Toll-like receptors sense danger signals known as PAMPs and DAMPs, causing neutrophil activation and neutrophil extracellular traps (NETs) to occur. These NETs and danger signals excessively activate the NLRP3 inflammasome, which in turn stimulates Caspase-1 to cleave pro-cytokines into their active, highly potent forms: IL-1β and IL-18. This initial cascade culminates in a self-sustaining "cytokine storm," a hallmark of the disease marked by the massive release of pro-inflammatory mediators including IL-6, IL-8, IL-17, TNF-α, and IFN-γ [3,5].

The transition to targeted biologic therapies has revolutionised the management of steroid-refractory AOSD. Recent phase III trials have shown the robust efficacy and safety profiles of interleukin inhibitors in this population. Specifically, the long-term extension of phase III trials has confirmed the sustained clinical efficacy and safety of the IL-6 inhibitor tocilizumab in maintaining disease remission [12]. Similarly, open-label phase III studies have validated the rapid efficacy and safety of the IL-1 inhibitor canakinumab, providing another highly effective, steroid-sparing option for patients presenting with severe autoinflammatory phenotypes [13]. 

## Conclusions

AOSD is a challenging diagnosis of exclusion that requires a high clinical suspicion in affected patients presenting with prolonged fevers, an evanescent rash, and striking hyperferritinaemia. Early recognition is imperative to prevent diagnostic delays and mitigate the risk of severe complications. While corticosteroids remain the cornerstone of initial therapy, the integration of targeted biologic agents, specifically IL-1 and IL-6 inhibitors such as anakinra and tocilizumab, can dramatically alter the disease trajectory. These therapies offer a highly effective, steroid-sparing approach that facilitates sustained remission and significantly improves the long-term prognosis for patients suffering from this severe autoinflammatory condition. Recent literature underscores the robust and sustained efficacy of these agents, while ongoing clinical trials continue to explore novel biologic targets and treatment combinations aimed at further reducing morbidity and mortality associated with AOSD. Future advances in disease-specific biomarkers and personalised immunotherapeutic strategies may enhance diagnostic precision and expand treatment options, ultimately transforming disease management and outcomes.
